# Statistical and spectral analysis of ECG signal towards achieving non-invasive blood glucose monitoring

**DOI:** 10.1186/s12911-019-0959-9

**Published:** 2019-12-19

**Authors:** Igbe Tobore, Jingzhen Li, Abhishek Kandwal, Liu Yuhang, Zedong Nie, Lei Wang

**Affiliations:** 1 Center for Medical Robotics and Minimally Invasive Surgical Devices, Shenzhen Institutes of Advanced Technology, Chinese Academy of Sciences, 1068 Xueyuan Avenue, Shenzhen University, Xili Town, Nanshan District, Shenzhen, China; 2 Graduate University, Chinese Academy of Sciences, Beijing, China

**Keywords:** Diabetes, Oral glucose tolerance test, ECG, Periodogram, Boxplot

## Abstract

**Background:**

Globally, the cases of diabetes mellitus (diabetes) have increased in the past three decades, and it is recorded as one of the leading cause of death. This epidemic is a metabolic condition where the body cannot regulate blood glucose, thereby leading to abnormally high blood sugar. Genetic condition plays a significant role to determine a person susceptibility to the condition, a sedentary lifestyle and an unhealthy diet are behaviour that supports the current global epidemic. The complication that arises from diabetes includes loss of vision, peripheral neuropathy, cardiovascular complications and so on. Victims of this condition require constant monitoring of blood glucose which is done by the pricking of the finger. This procedure is painful, inconvenient and can lead to disease infection. Therefore, it is important to find a way to measure blood glucose non-invasively to minimize or eliminate the disadvantages encountered with the usual monitoring of blood glucose.

**Method:**

In this paper, we performed two experiments on 16 participants while electrocardiogram (ECG) data was continuously captured. In the first experiment, participants are required to consume 75 g of anhydrous glucose solution (oral glucose tolerance test) and the second experiment, no glucose solution was taken. We explored statistical and spectral analysis on HRV, HR, R-H, P-H, PRQ, QRS, QT, QTC and ST segments derived from ECG signal to investigate which segments should be considered for the possibility of achieving non-invasive blood glucose monitoring. In the statistical analysis, we examined the pattern of the data with the boxplot technique to reveal the change in the statistical properties of the data. Power spectral density estimation was adopted for the spectral analysis to show the frequency distribution of the data.

**Results:**

HRV segment obtained a statistical score of 81% for decreasing pattern and HR segment have the same statistical score for increasing pattern among the participants in the first quartile, median and mean properties. While ST segment has a statistical score of 81% for decreasing pattern in the third quartile, QT segment has 81% for increasing pattern for the median. From a total change score of 6, ST, QT, PRQ, P-H, HR and HRV obtained 4, 5, 4, 5 and 6 respectively. For spectral analysis, HRV and HR segment scored 81 and 75% respectively. ST, QT, PRQ have 75, 62 and 68% respectively.

**Conclusions:**

The results obtained demonstrate that HR, HRV, PRQ, QT and ST segments under a normal, healthy condition are affected by glucose and should be considered for modelling a system to achieve the possibility of non-invasive blood glucose measurement with ECG.

## Introduction

Diabetes mellitus (diabetes) is a metabolic disorder with global health threat, because it affects both the young and old. According to International Diabetes Federation (IDF), an estimation of 1 in 2 adults is diagnosed with diabetes (212 million) and 1 in 11 adults aged 20–79 years (425 million) had diabetes mellitus globally in 2017 [1]. Also, this estimate is expected to increase to about 642 million by 2040, and the largest increases will come from the regions experiencing economic transitions from low-income to middle-income levels [2]. Diabetes arises when the body is unable to control or balance the concentration of blood glucose (BG), there are several reasons for the development of this epidemic. This could be as a result of autoimmune destruction of the β-cells of the pancreas with consequent deficiency of insulin or abnormalities which causes resistance to insulin action in the body tissues and organs [3]. Some factors contribute to the epidemic of diabetes, including urbanization, population ageing, economic development, unhealthy eating habit and sedentary lifestyles [2].

The intake of glucose is part of everyday meals for humans and it is considered as the clinical assessment of glucose tolerance by a method called oral glucose tolerance test (OGTT). In this method, BG value is measured after an overnight fast/or 2 h after ingesting 75 g of glucose solution. An individual has been classified as having diabetes based on the results of this method. Impaired glucose tolerance, defined as 2-h oral glucose tolerance level of 7.8–11.0 mmol/dl, and impaired fasting glucose, defined as fasting glucose level of 6.1–6.9 mmol/dl, according to World Health Organization (WHO) criteria [4]. Although, the recommendation of 2-h OGTT by WHO is regarded as the best choice, however, due to economic or logistic reasons, fasting glucose alone may be considered for the classification. There is a common occurrence of abnormal high BG in diabetes, sufficient to cause pathologic and functional changes in various tissues, but without clinical symptoms, this may be present for a period of time before diabetes is detected [5]. Victims of diabetes suffer from one or more complications which include retinopathy with a potential loss of vision; nephropathy leading to renal failure; peripheral neuropathy with risk of foot ulcers, amputations, and Charcot’s joints; and autonomic neuropathy causing gastrointestinal, genitourinary, and cardiovascular symptoms and sexual dysfunction [5]. Patients with diabetes have an increased incidence of atherosclerotic cardiovascular, peripheral arterial, and cerebrovascular disease [5, 6]. Hypertension and abnormalities of lipoprotein metabolism are often found in people with diabetes [5, 7].

Unfortunately, there is no treatment for this disorder, patients must monitor their BG concentration and administer insulin to keep BG values within a desirable range of 70–180 mg/dL. The over administration of insulin can lead to low blood glucose (hypoglycemia), while failure to administer enough insulin can lead to high blood glucose (hyperglycemia) [7]. Conventionally, patients draw blood by pricking the finger and with the help of a strip and portable device, the blood is analyzed to know the glucose level. This technique can be painful because the patient is required to do this multiple times in a day. Also, with the use of the needle for pricking the finger, this can cause irritation, contamination and disease transmission. Non-invasive techniques would be an appropriate solution. In [8], a bioimpedance difference considering blood volume pulsation was presented using inhomogeneous arm model to achieve non-invasive BG monitoring. Transcutaneous approach to BG monitoring has been considered [9]. The system consists of a suction apparatus and the glucose sensor system. A vacuum is applied to the patient’s skin at 400 mmHg absolute pressure to collect the suction effusion fluid (SEF). Miniature ion-sensitive transistor-based glucose sensor is used to measure glucose in small SEF quantities. Other proposed methods include: infrared/near-infrared spectroscopy [10, 11], optical sensing [12], Raman spectroscopy [13], microwave spectrum [14, 15], capacitance measurement technique [16], skin conductance [17, 18]. There are significant promising results that have been achieved with the use of these methods. However, the use of physiological signal such as an electrocardiogram (ECG) can provide an alternative approach to achieve the possibility of non-invasive measurement of BG.

Moreover, there is evidence that hypoglycemia can affect ECG segments, such as prolongation of QT interval [19], and increase heart rate [20]. In [21], Multi-Layer Perceptron (MLP) Classification of hypoglycemia using five ECG features was presented namely: RR, RTc, T wave amplitude, T wave skewness and T wave kurtosis. Also in [21], a rule base method with Two ECG features: T wave amplitude and RTc was combined for monitoring hypoglycemia. A combination of corrected T interval together with QTc and heart rate was considered for detection of hypoglycemia [22]. The analysis in [23], proved that QTc, QTc dispersion and PR interval have a significant change to hyperglycemia condition. In their experiment, plasma glucose concentrations were raised to 15 mmol/l in 20 healthy subjects. Systolic and diastolic blood pressures, heart rate and plasma catecholamine concentrations showed significant increase which started after 60 min of hyperglycemia. Consequently, heart rate (HR), heart rate variability (HRV) and baroreflex sensitivity were compared for hyperglycemia prediction [17]. Acquisition and analysis based on LabVIEW were developed for identification of hypoglycemia and hyperglycemia [24]. In the analysis, the following ECG parameters were considered: Heart rate, corrected QT interval, PR interval, corrected RT interval and corrected TpTe interval. These studies have used different features from ECG signal and reported promising results with a different technique. However, it is necessary to investigate ECG segments that are affected by a change in BG that should be considered to achieve the possibility of non-invasive blood glucose monitoring.

In this study, we investigated two methods to discover the segments in the ECG signal that can be useful for non-invasive blood glucose measurement. OGTT experiment was conducted on 16 volunteers, while ECG signal was continuously recorded for a period of 2 h. Then, we applied spectral and statistical analysis to observe the pattern in nine ECG segments.

## Methods

In this section, we describe the statistical analysis and spectral analysis of the ECG signal to observe the change in ECG segments to change in BG. We begin by discussing the implementation of OGTT experiment and the simultaneous acquisition of the ECG signal. Then we examine nine segments extracted from the captured ECG signal to provide information about how glucose affects the ECG waveform. This will provide evidence for the possibility of achieving non-invasive blood glucose monitoring.

### Experiment and data

**Fig. 1:**
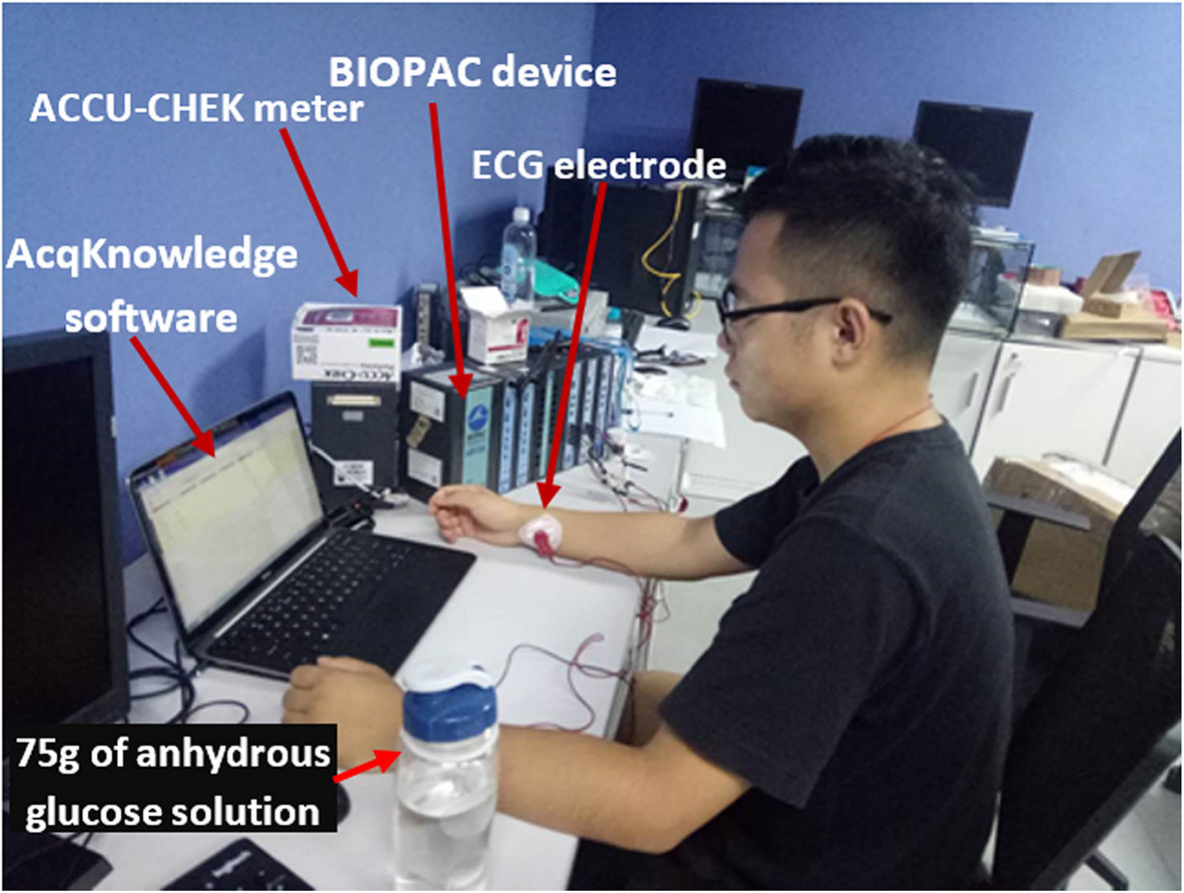
Participant during the OGTT experiment with connected one channel ECG electrodes. Participant gave their permission to publish their image

The experiment to uncover the effect of BG on the ECG signal is investigated with OGTT on 16 participants. The setup for the experiment requires the participants to sit in a comfortable position for a period of 120 min. Single-channel ECG electrodes are connected to the patient on the arms (positive and negative electrodes) and on the leg (ground electrode). Figure 1 shows a participant during the experiment, all participants that participated in the experiment signed the consent form approved by Internal Review Board (IRB) of Shenzhen Institute of Advanced Technology (SIAT), Chinese Academy of Sciences (SIAT-IRB-181015-H0266). A day before the experiment, participants are informed to eat dinner early in the evening and on the day of the experiment, breakfast should not be taken. The experiment begins at about 8:00 am and 30 min into the experiment, participant is required to ingest a prepared 75 g of anhydrous glucose solution. There are 10 males and 6 females that participated in the experiment, their age ranges between 24 and 35, bodyweight is between 48 kg and 96 kg. Their height is between 1.60 m and 1.96 m, they have constant normal body temperature and regular heart rate throughout the experiment.

Continuous ECG signal is captured with a stacked BIOPAC device (model number: MP150) and AcqKnowledge software. The electrode from the participant is connected to the BIOPAC device, which converts the analogue ECG signal into a digital value for the computer. AcqKnowledge software in the computer was responsible for receiving and storing the digital signal. Also, the software has the feature to annotate and extract segments from the ECG signal and save it as comma-separated value (csv) file in the computer. To compare the changes in ECG data, each participant is required to participate in the experiment twice for 2 days consecutively. On the first day, participants are required to consume glucose solution while ECG is continuously captured. On the second day, the experiment is repeated but glucose solution is not given to the participant, this serves the control for the experiment that was performed on the first day. Therefore, the first day is referred to as glucose (G) experiment and the second day is the non-glucose (NG) experiment. Throughout the experiment for both days, at 25 min interval the BG level was measured by pricking the finger and the value was determined with a blood strip device called ACCU-CHEK meter.

### ECG segments

**Fig. 2:**
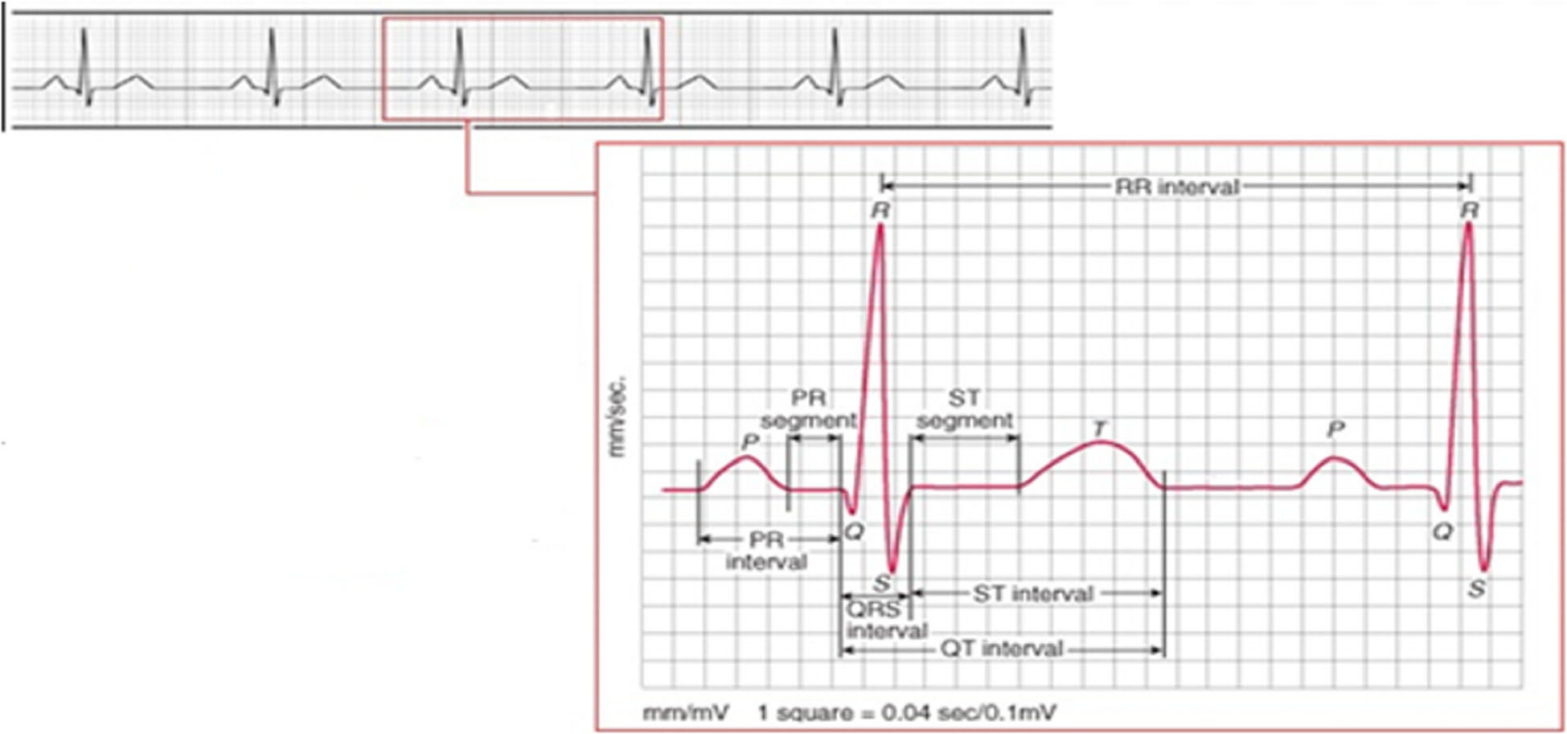
Description of constituent segments of a complete ECG waveform for a cardiac cycle

In a complete successive ECG waveform, nine segments can be extracted, which represents the characteristics of a cardiac cycle. Figure 2 shows a description of a single ECG signal and the segments derived from the waveform. Important points are marked with alphabet ‘P’, ‘Q’, ‘R’, ‘S’, ‘T’ and ‘U’ for reference and description. Measurements are taken between these points to reveal the properties and status of the waveform; these values are either positive or negative depending on the shape of the signal or measurement under consideration. The nine ECG segments are described in Table 1, we considered all the segments in our analysis to discover the behaviour concerning glucose consumed. The ECG signal captured during G and NG experiment is analyzed after extracting the segments. The following are the extracted ECG segments: RR-I (HRV), HR, R-H, P-H, QRS, PRQ, QT, QTC and ST.

**Table 1:** ECG segments from a complete ECG waveform

Number	Parameter	Meaning
1	RR-I (HRV)	RR interval is the interval between successive Rs of the ECG wave and R is a point located at the peak of the QRS complex (Heart rate variability (HRV))
2	HR	Heart rate computed in beat per minutes
3	R-H	Height of the R wave in millivolts
4	P-H	Height of the P wave in millivolts
5	QRS	Time interval of the QRS complex
6	PRQ	The interval from the beginning of the P wave to the peak of R wave in milliseconds
7	QT	QT segment interval in millisecond
8	QTC	Corrected QT intervals
9	ST	ST interval in milliseconds after the S wave to the beginning of the T wave

*ECG* Electrocardiogram

### Statistical analysis

To observe the behaviour and characteristics of data from G and NG experiment, a boxplot is created to represent the distribution of the values in the extracted ECG segments. Boxplot is a graphical representation of the distribution of data that consists of five statistical characteristics: maximum value, third quartile (*Q*3), median (*Q*2), first quartile (*Q*1) and minimum values. In this analysis, we did not consider the maximum and minimum values because they can easily be affected by noise and other external factors. However, we considered the mode (most occurring) value from the ECG segments. The following equation describes the statistical analysis performed on the ECG data.

1 $$ \Delta  md\left[i\right]= md{\left[i\right]}_g- md{\left[i\right]}_{ng} $$

2 $$ \Delta  mn\left[i\right]= mn{\left[i\right]}_g- mn{\left[i\right]}_{ng} $$

3 $$ \Delta  mdn\left[i\right]= mdn{\left[i\right]}_g- mdn{\left[i\right]}_{ng} $$

4 $$ \Delta  Q3\left[i\right]=Q3{\left[i\right]}_g-Q3{\left[i\right]}_{ng} $$

5 $$ \Delta  Q1\left[i\right]=Q1{\left[i\right]}_g-Q1{\left[i\right]}_{ng} $$

6 $$ \Delta  IQ{\left[i\right]}_{ng}=Q3{\left[i\right]}_{ng}-Q1{\left[i\right]}_{ng} $$

7 $$ \Delta  IQ{\left[i\right]}_g=Q3{\left[i\right]}_g-Q1{\left[i\right]}_g $$

8 $$ \Delta  Q\left[i\right]=\Delta  IQ{\left[i\right]}_g-\Delta  IQ{\left[i\right]}_{ng} $$

Where *md*[*i*]_*g*_ and *md*[*i*]_*ng*_ are the mode values for G and NG data obtained from segment *i* (1 ≤ *i* ≤ 9) and the difference is represented as *∆md*[*i*]. For each ECG segment, *mn*[*i*]_*g*_ and *mn*[*i*]_*ng*_ are the mean value *mdn*[*i*]_*g*_ and *mdn*[*i*]_*ng*_ are the median value, *∆mn*[*i*] and *∆mdn*[*i*] represents the difference in mean and median values respectively. *∆Q*3[*i*] and *∆Q*1[*i*] are the difference between the third and first quartile respectively, the interquartile range for N and NG are represented as *∆IQ*[*i*]_*g*_ and *∆IQ*[*i*]_*ng*_ respectively. *∆Q*[*i*] is the difference between the interquartile range (IRQ) from G and NG data from ECG segments.

To investigate the interdependence relationship between the ECG segments for G experiment, multivariate analysis using the correlation coefficient is computed between the segments. The purpose is to reveal the connection between the segments to know how different segments are affected during the experiment. The computation of the correlation is achieved with the following equation:

9 $$ {R}_{a^i{b}^i}=\frac{n\sum {a}^i{b}^i-\sum {a}_j^i\sum {b}_j^i}{\sqrt{n\sum {a}_j^{i^2}-{\left(\sum {a}_j^i\right)}^2}\sqrt{n\sum {b}_j^{i^2}-{\left(\sum {b}_j^i\right)}^2}} $$

Where ${R}_{a^i{b}^i}$ is the result of the correlation between two segments from ECG waveform from the glucose experiment, the value can either be equal to 0, greater than 0 or less than 0 which represents no correlation, positive correlation and negative correlation respectively. The description of correlation and representation is presented in Table 2. *a*^*i*^ and *b*^*i*^ are ECG segments, ${a}_j^i$ and ${b}_j^i$ represents values in the segments respectively.

**Table 2:** ECG segments correlation representation

Condition	Expression	Pattern representation
Positive correlation	${R}_{a^i{b}^i}>0$	Positive (P)
Negative correlation	${R}_{a^i{b}^i}<0$	Negative (N)
No (Zero) correlation	${R}_{a^i{b}^i}=0$	Zero (Z)

### Spectral analysis

Statistical signal analysis is designed to observe the difference between G and NG ECG segment data with a different analysis technique. We perform spectral analysis by computing spectral density estimation. The purpose is to investigate each ECG segments in frequency domain using power spectral density (PSD) formula to reduce the variance of the distribution in each ECG segment data. The density estimation is based on the periodogram spectrum estimate, which is achieved by converting the data in each segment from the time domain to frequency domain. An improved estimator is provided by welch method [25]. The data is first divided into overlapping partitions, then the periodogram of each partition is computed before calculating the power density estimate [26]. The following equation is used to compute the periodogram for each ECG segment data given by *D*;

10 $$ D{\left[i\right]}_m(n)=w(n)D\left[i\right]\left(n+ mR\right) $$

where *D*[*i*] represents one ECG segment and *n* = 0, 1, 2, …, *M* − 1. where *m* = 0, 1, …, *K* − 1 and *k* denote the number of frames, *w*(*n*) is the rectangular window and *R* is the step size for overlap and *D*[*i*]_*m*_(*n*) is the *m* th windowed frame from *D*[*i*]. Therefore, the periodogram of the *m* th block, ${P}_{D{\left[i\right]}_m,M}\left({\omega}_k\right)$ is given by

11 $$ {P}_{D{\left[i\right]}_m,M}\left({\omega}_k\right)=\frac{1}{M}{\left|\sum \limits_{n=0}^{N-1}D{\left[i\right]}_m(n){e}^{-i2\pi nk/N}\right|}^2 $$

The average of the periodograms across time, *S*_*D*_(*ω*_*k*_), which is the welch method is denoted by:

12 $$ {S}_{D\left[i\right]}^y\left({\omega}_k\right)=\frac{1}{K}\sum \limits_{m=0}^{K-1}{P}_{D{\left[i\right]}_m,M\left({\omega}_k\right)} $$

Where *y* in ${S}_{D\left[i\right]}^y$ can either be G or NG which represents glucose and non-glucose data from *D*[*i*].

The pattern of change from this analysis is represented in direction, where the direction of change is said to be increased if the analysis parameter value in G > NG, or decrease if G < NG and no change is recorded if G = NG. Also, the peak value between G and NG from the periodograms are compared to determine the direction of change, which can either be an increase, decrease or equality based on the condition presented in Table 3. The function *H* in the table obtains the peak value from ${S}_{D\left[i\right]}^y$.

**Table 3:** Description and color representation for changes in statistical and spectral analysis

Statistical parameter (STP)	Spectral parameter (SPP)	Direction	Pattern
{*∆md*[*i*], *∆mn*[*i*], *∆mdn*[*i*], *∆Q*3[*i*], *∆Q*1[*i*], *∆Q*[*i*] } > 0	$H\left({S}_{D\left[i\right]}^G\right)>H\left({S}_{D\left[i\right]}^{NG}\right)$	Increase	I
{*∆md*[*i*], *∆mn*[*i*], *∆mdn*[*i*], *∆Q*3[*i*], *∆Q*1[*i*], *∆Q*[*i*]} < 0	$H\left({S}_{D\left[i\right]}^G\right)<H\left({S}_{D\left[i\right]}^{NG}\right)$	Decrease	D
{*∆md*[*i*], *∆mn*[*i*], *∆mdn*[*i*], *∆Q*3[*i*], *∆Q*1[*i*], *∆Q*[*i*]} = 0	$H\left({S}_{D\left[i\right]}^G\right)=H\left({S}_{D\left[i\right]}^{NG}\right)$	Equality	E

We compute the percentage change in direction, *d*_*z*_% as:

13 $$ {d}_z\%=\frac{\sum {d}_z}{\sum d}\times 100 $$

Where *d*_*z*_ represents the change in a specific direction, z can either be 1, 2, 3 representing an increase, decrease, equality respectively and *d* represents the total observable change from the analysis. Therefore, the status of *d*_*z*_% can be computed with the following criteria

14 $$ status=\Big\{{\displaystyle \begin{array}{ll}100\ge {d}_z\%\ge 80& (h)\\ {}80>{d}_z\%\ge 60& (m)\\ {} else& (l)\end{array}} $$

Where (*h*) status represents a strong occurring pattern among the participants, (*m*) is moderate status which signifies pattern that occurs in above-average number of the participants and every other value is represented as (*l*) which is means low status, a weak pattern.

Finally, we compute the sensitivity of each segment from the status with the following equations:

15 $$ {S}_h\left[i\right]=\frac{\sum status\left[i\right]=(h)}{\sum status\left[i\right]}\times 100\% $$

16 $$ {S}_m\left[i\right]=\frac{\sum status\left[i\right]=(m)}{\sum status\left[i\right]}\times 100\% $$

17 $$ {S}_l\left[i\right]=\frac{\sum status\left[i\right]=(l)}{\sum status\left[i\right]}\times 100\% $$

Where *status*[*i*] is the status label for a particular ECG segment and *S*_*h*_[*i*], *S*_*m*_[*i*] and *S*_*l*_[*i*] are the sensitivity of the status label (*h*), (*m*) and (*l*) respectively for a particular ECG segment.

## Results and discussion

**Fig. 3:**
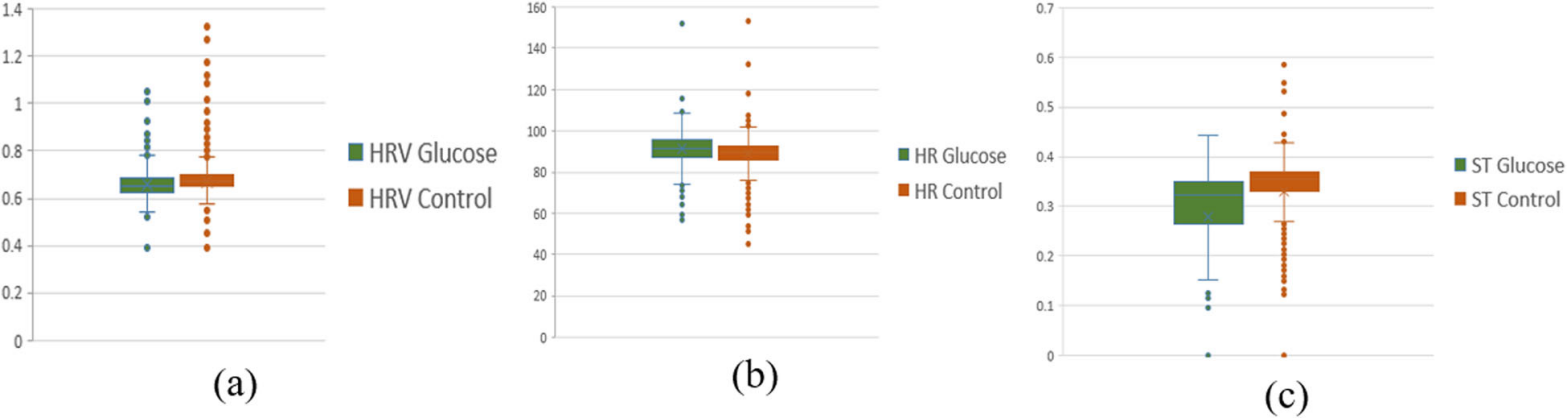
Boxplot from one participant for **a** HRV **b** HR **c** ST ECG segments

Figure 3 shows the boxplot of G and NG experiment for three ECG segments from one of the participants. The data collected during the experiment from each participant have an average of 13,168 cycles of the ECG signal. The blood glucose value recorded from the participants during the OGTT experiment ranges from 4.8 mmol/dl to 12 mmol/dl for 4 of the participants and 4.5 mmol/dl to 10 mmol/dl for 12 of the participants. BG value during the control experiment is between 4.8 mmol/dl and 5.5 mmol/dl for all participants.

**Fig. 4:**
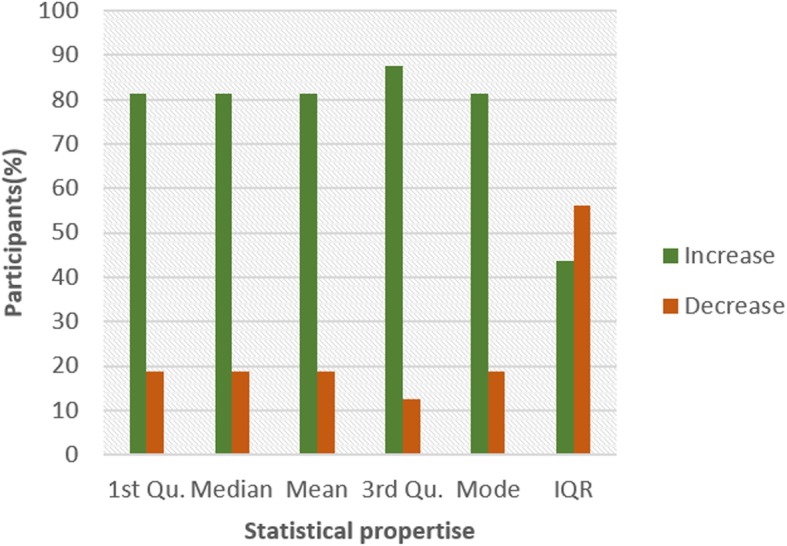
Pattern of HR segment from ECG signal from G and NG experiment

**Fig. 5:**
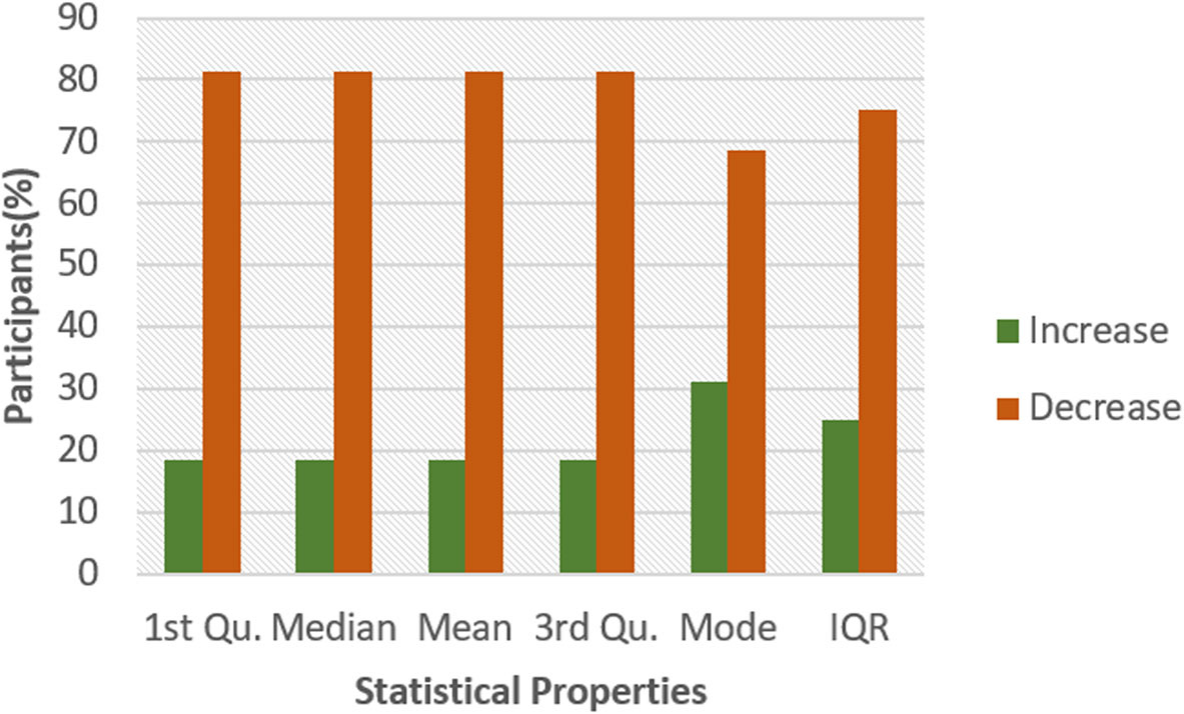
Pattern of HRV segment from ECG signal from G and NG experiment

Figures 4 and 5 show the pattern for HR and HRV ECG segment data respectively from all the participants. The figure shows the colour code representing the pattern of change between N and NG experiments for the statistical properties. A general examination of the results reveals that the influence of glucose has a significant effect on both segments. In Fig. 4, it can be seen that there is an increasing pattern in the statistical properties of HR segment in most of the participants. However, there are few exceptions with less than 20% that have a decreasing pattern. This exceptional behaviour is found in participant 2 which shows a reduction pattern for first quartile, median and mode. Participants 10 and 13 also have a similar pattern with participant 2. Also, third quartile and mode pattern also have an increasing pattern. The deviation from the regular pattern observed in participants 2, 10 and 13 requires further investigation to identify the reason for the difference in behaviour. The mode pattern for participant 9 has an increasing pattern, however, the difference in the value is small (0.58) compared to the values available in the segments. There is no convincing pattern in the inter-quartile range because 56% of the participants have a decreasing pattern and 44% have an increasing pattern. Furthermore, the behaviour of the pattern in Fig. 5 is the opposite of the pattern in Fig. 4. Most of the participants have a decreasing pattern for the statistical parameters. Again, there are few exceptions which are found in participant 2, 10 and 13 that shows an increasing pattern for median, mean, third quartile and mode. While participant 10, 11 and 13 have an increasing pattern for the first quartile, participant 6 and 9 have an increasing pattern for mode. However, the deviation from the regular pattern is more conspicuous for participant 2, 10 and 13. This requires further study to uncover the reason for the deviation of these participant’s characteristics to change in BG. HR and HRV response to glucose have an indirect change pattern with one another, HR and HRV segment score 81% increasing pattern and decreasing pattern respectively in most of the statistical properties.

**Fig. 6:**
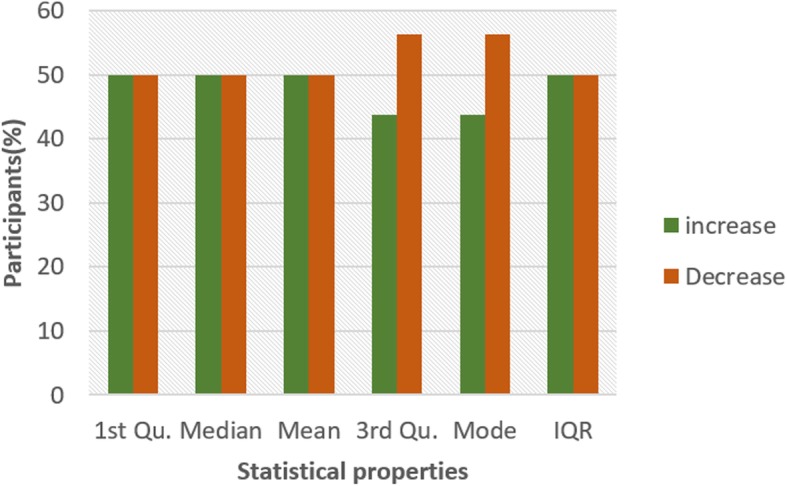
Pattern of R-H segment from ECG signal from G and NG experiment

**Fig. 7:**
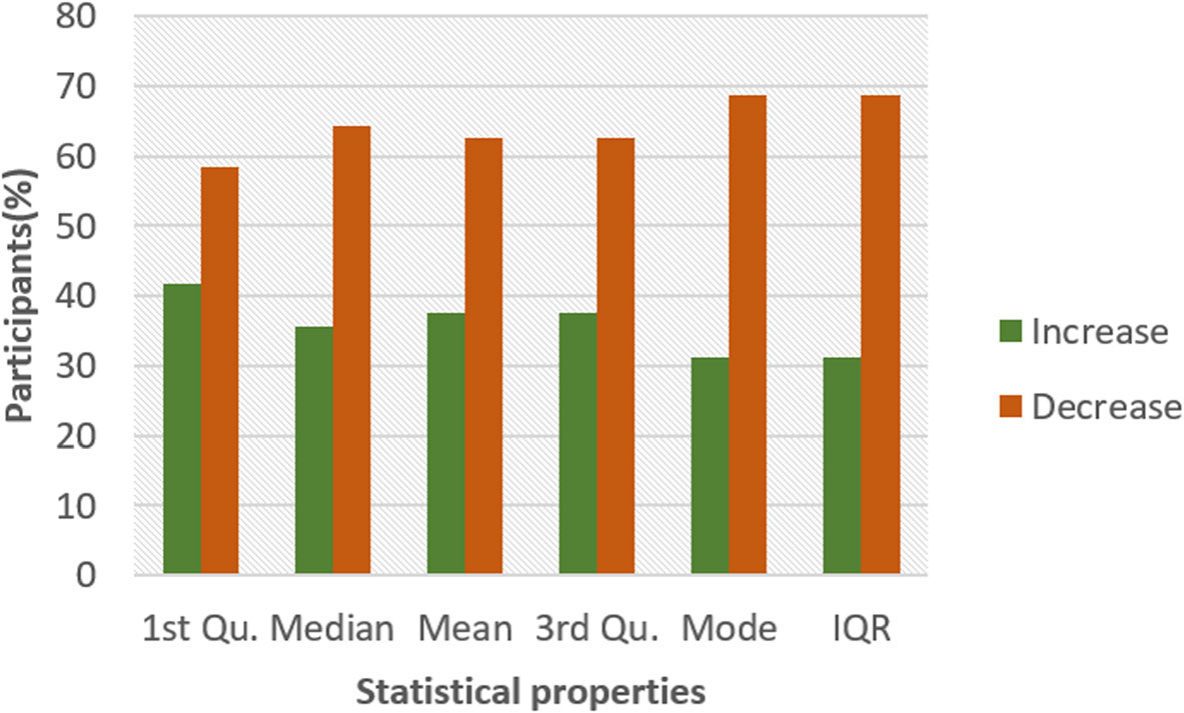
Pattern of P-H segment from ECG signal from G and NG experiment

**Fig. 8:**
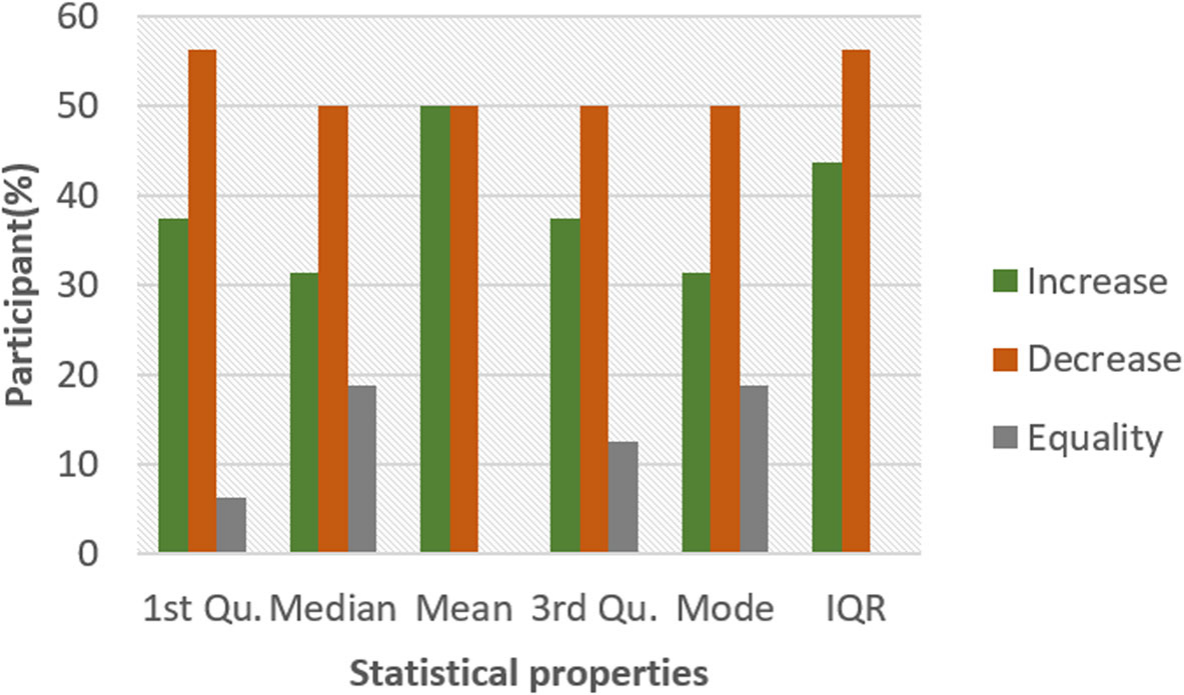
Pattern of QRS segment from ECG signal from G and NG experiment

**Fig. 9:**
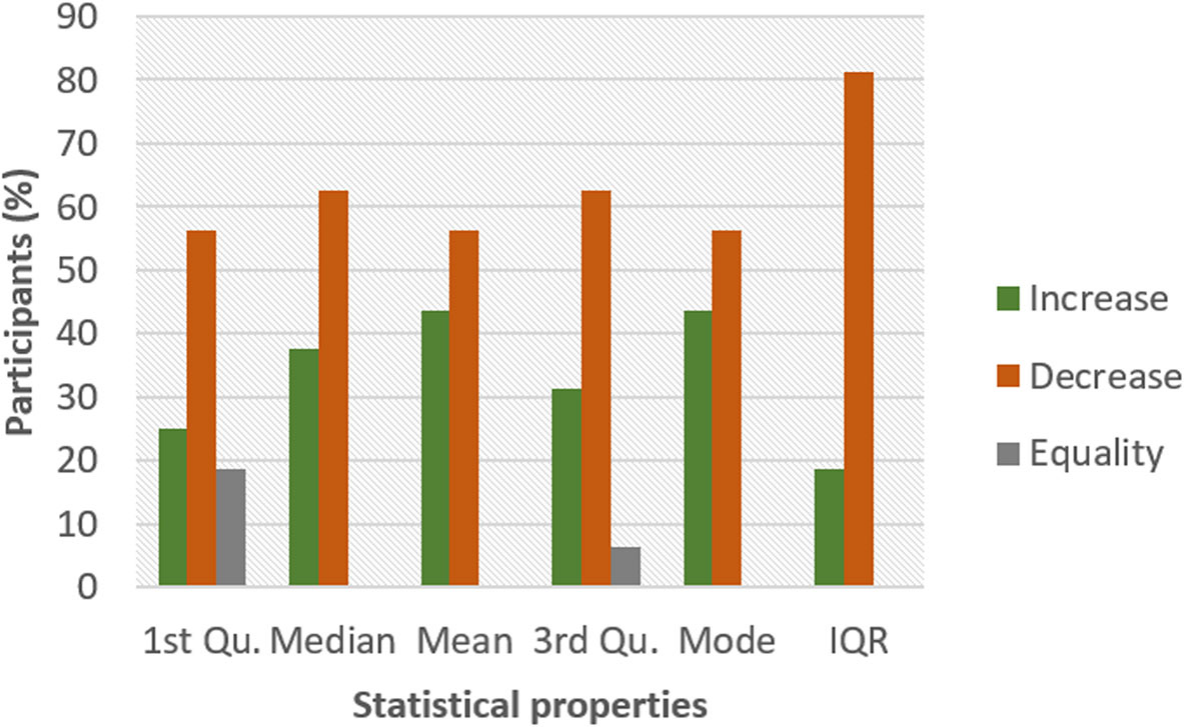
Pattern of PRQ segment from ECG signal from G and NG experiment

**Fig. 10:**
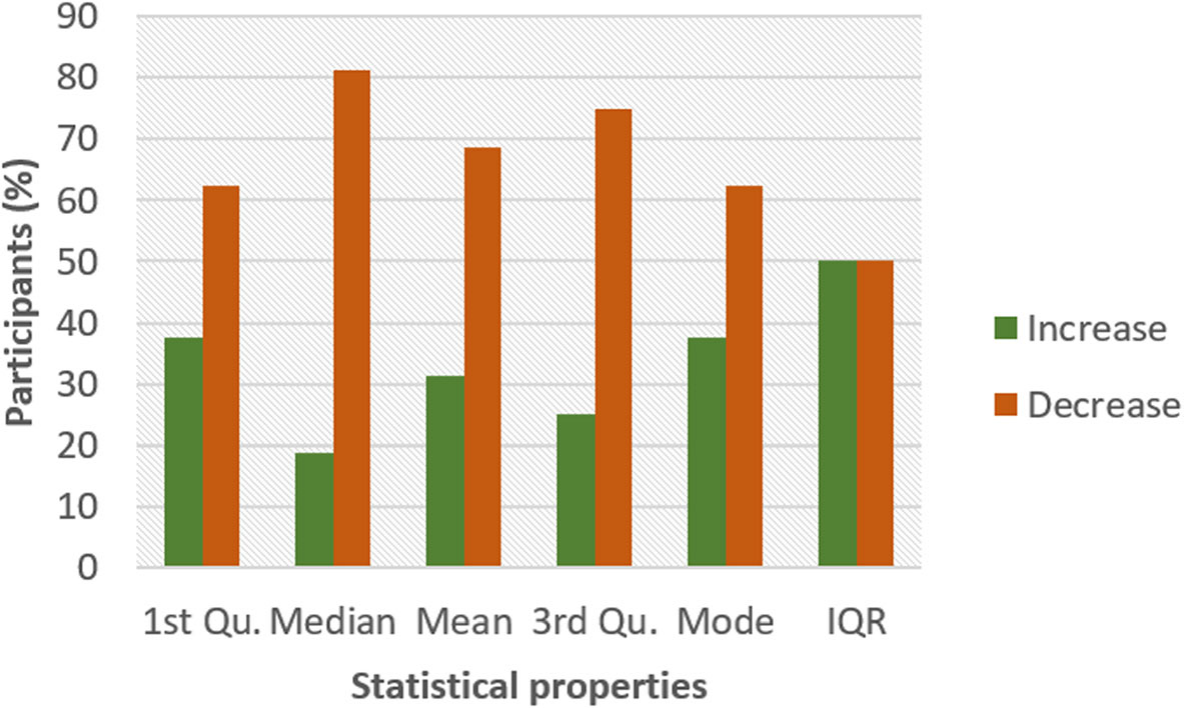
Pattern of QT segment from ECG signal from G and NG experiment

**Fig. 11:**
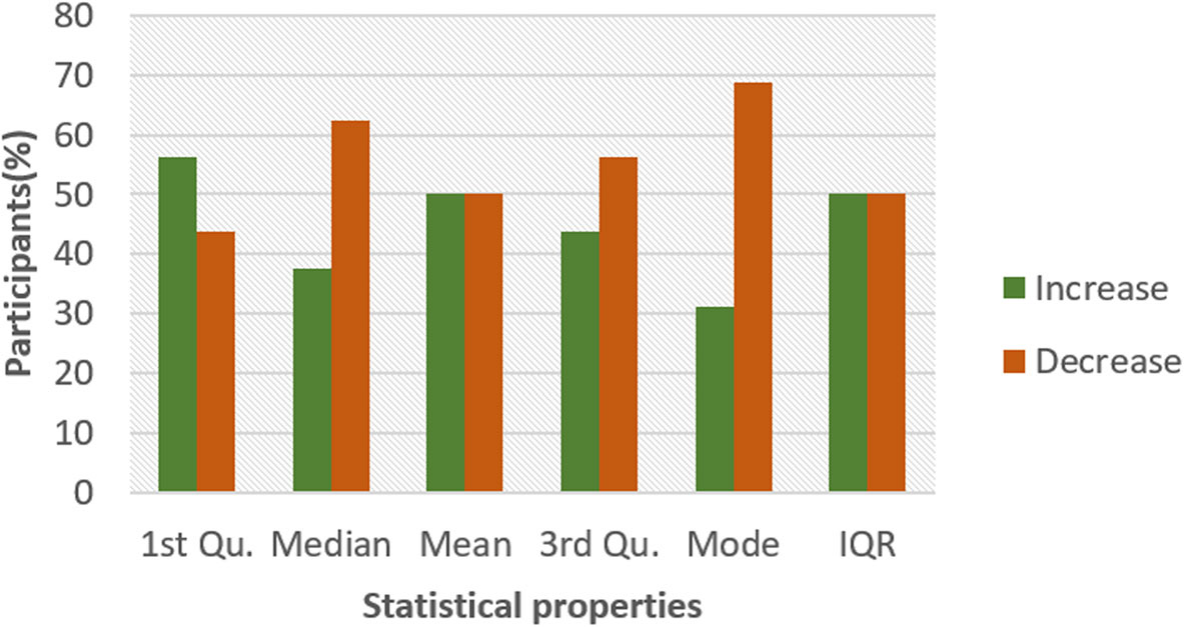
Pattern of QTC segment from ECG signal from G and NG experiment

**Fig. 12:**
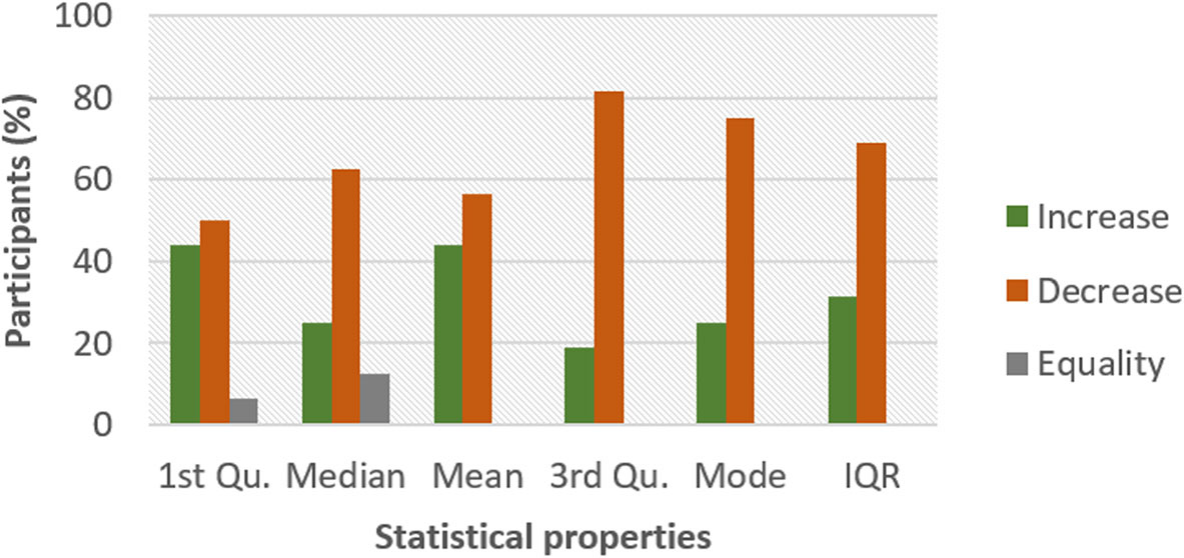
Pattern of ST segment from ECG signal from G and NG experiment

To investigate the influence of glucose on other ECG segments, Figs. 6, 7, 8, 9, 10, 11, and 12 describes the pattern of the statistical parameters among the participants for the other ECG segments. ST and QT segments in Figs. 10 and 12 respectively have patterns that reveal a common behaviour among the participants. Where *∆Q*3 and *∆md* values in ST segment shows a decreasing pattern with 81 and 75% respectively. While for the QT segment, the value for *∆mdn* and *∆Q*3 are 81 and 75% respectively for decreasing pattern. However, 19% of *∆mdn* in QT segment and *∆Q*3 in ST segment have a different behaviour that shows an increasing pattern. The percentage value of *∆Q* for PRQ shows a decreasing pattern with a value of 81%. This means that the width of the distribution is clustered around the central value for most of the participants. Another interesting pattern is found in R-H and QTC segments, while for R-H segment the percentage value of 50% for an increase and decrease pattern is found in the statistical properties except for *∆Q*3 and *∆md*. Similarly, in QTC segment the difference between the increase and decrease pattern is not far from each other and it is closely similar to the R-H segment. This means a further study of these segments can provide useful information that can be exploited to divide participants into clusters to create a model to distinguish participants with similar features. Moreover, HR and HRV segments have a more consistent pattern compared to other segments. Therefore, from the results, we can say that the following ECG segments: HR, HRV, ST and QT exhibit behaviours that show significant changes for BG. Also, with further investigation R-H, QTC and PRQ segment can be included due to the pattern present in the features.

To summarize the results from Figs. 4, 5, 6, 7, 8, 9, 10, 11 and 12, we counted the occurrence of the status (*m*) and (*h*) in each ECG segment from all the participants. Table 4 represents the result of the occurrence of change in status. Therefore, from the statistical analysis, to determine if a particular ECG segment is affected by glucose the total occurrence score of the status is set to be ≥4, where the maximum obtainable score is 6. The label ‘Yes’ value in the change column in the table means the segment is affected by BG and ‘No’ means the segment is unaffected by BG based on the total occurrence score. Therefore, the following ECG segments are affected by glucose: ST, QT, PRQ, P-H, HR and HRV as shown in the table.

**Table 4:** Summary of occurrence for change in status

ECG	Status		Total	Change
	(*m*)	(*h*)		
ST	3	1	4	Yes
QTC	2	0	2	No
QT	4	1	5	Yes
PRQ	3	1	4	Yes
QRS	0	0	0	No
P-H	4	0	4	Yes
R-H	0	0	0	No
HR	0	5	5	Yes
HRV	2	4	6	Yes

(*m*): Low

(*h*): High

*ECG* Electrocardiogram

The outcome of the correlation between the ECG segments is presented (see Additional file 1). ST, QTC and QT are represented by *a*^*i*^ and PRQ, QRS, P-H, R-H, HR, HRV are represented by *b*^*i*^. We want to investigate the combination of QTC segment with HR and HRV because QTC segment shows a low change according to Table 4. Then, QRS and R-H segments with ST, QTC and QT segments. Also, to investigate the combination of ST, QT segments with HR and HRV segments because they both have good results in the univariate analysis. The correlation percentage is computed by multiplying the ratio of the total positive or negative correlation outcome by the total number of observations with 100. The result shows that HRV is 92% positively correlated with ST and QT segments while HR is 92% negatively correlated for the same segments. This shows that HRV and HR combined with ST and QT segments are the favorable combinations that should be considered for the possibility to achieve BG monitoring. For the QTC segment, HR is 62% positively correlated while it is 62% negatively correlated for HRV segment. PRQ and QRS segments are 69% negatively correlated, although PRQ is 85% negatively correlated with QTC, QRS is 77% positively correlated with QTC. Besides HRV, HR and QRS segments that show consistent high correlation values, PRQ segments have a good relationship with ST, QT and QTC segments. P-H and R-H have the least correlation percentage value and should be least considered for non-invasive BG monitoring.

**Fig. 13:**
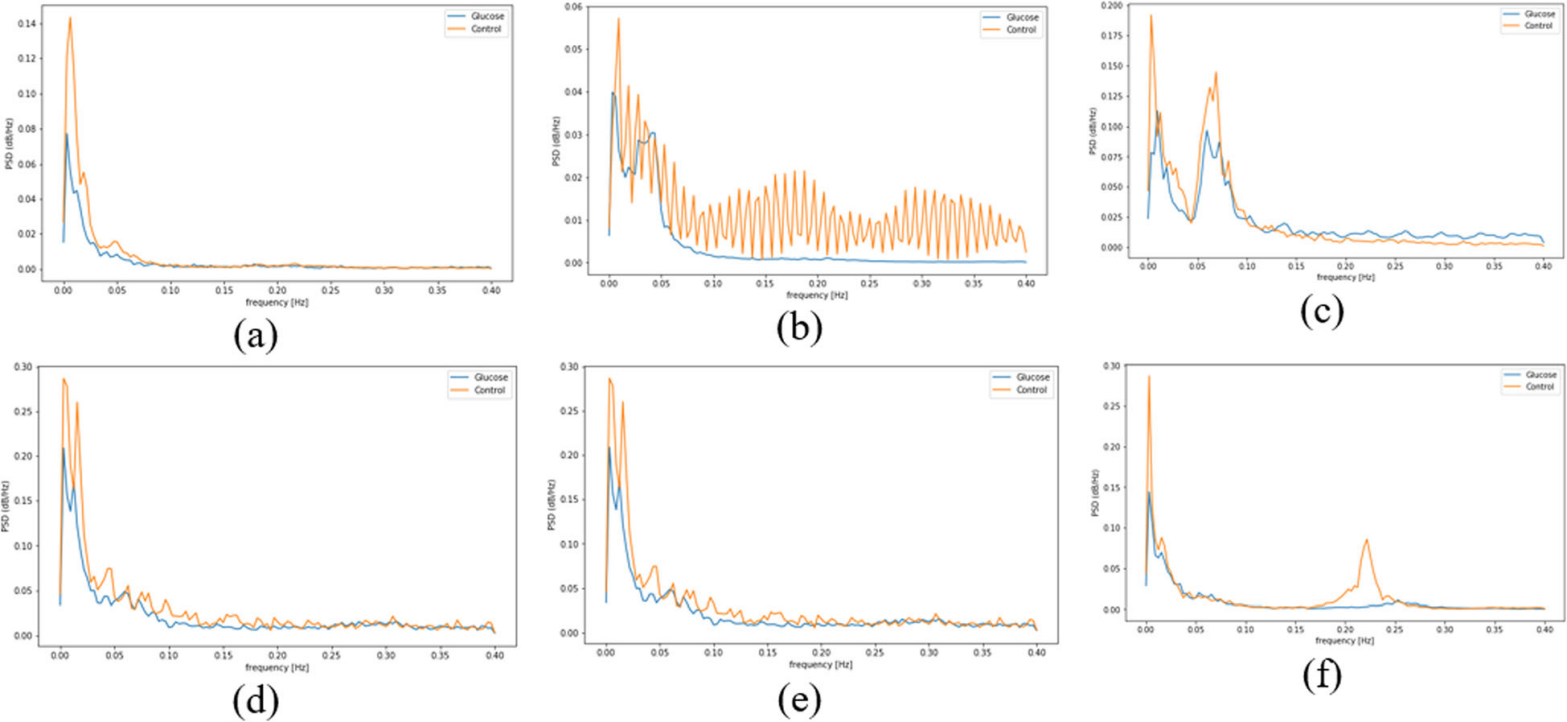
PSD graph for HRV ECG data for participant **a** 1 **b** 2 **c** 3 **d** 4 **e** 5 **f** 6

The creation of the PSD from the ECG segment data is done by setting the sampling frequency value at 0.8 to focus on the peak values in the data, the length of each frame is set at 256 to hold as much data in a frame and the number of points to overlapping between the frame is set at 10. Figure 13 shows the diagram of the PSD graph for the frequency distribution from six participants for HRV segment. The blue line in the graph represents data from G experiment and the yellow line is the data from NG experiment. The percentage difference between the peak value from both experiment is presented in Table 5. HRV and HR segments have the highest percentage of change pattern with 81 and 75% respectively. This result is similar to the outcome from the statistical analysis, this further proves that these segments are affected by blood glucose. However, the small percentage that shows a different pattern needs to be investigated to understand the reason for the deviation. Furthermore, ST, QT and PRQ have moderate *(m)* pattern with a score of 75, 62 and 68% respectively. These segments can be combined with HRV and HR segment for the possibility of achieving non-invasive blood glucose monitoring. Moreover, it is important to consider how these segments are combined because of the variation observed in some participants. The segments with status (*l*) are: QTC, QRS, P-H and R-H because their change in pattern score is less than 60%. Also, the result of R-H from Table 5 is similar to Fig. 6 because the percentage pattern closely divides the participants into two halves. For the spectral analysis, we set a threshold of ≥60% for pattern occurrence percentage to determine if a segment is affected by glucose. The ‘Yes’ in the change column indicate that it is affected by glucose and ‘No’ mean it is not affected by glucose. Therefore, the following ECG segments are affected by glucose: ST, QT, PRQ, HR and HRV. These affected segments are similar to the segments obtained from statistical analysis except for the absence of P-H segment.

**Table 5:** Percentage occurrence for the pattern of peak value from PSD

Segments	Pattern	Occurrence	Status	Change
ST	Decrease	75%	(m)	Yes
	Increase	25%	(l)	
QTC	Decrease	56%	(l)	No
	Increase	44%		
QT	Decrease	62%	(m)	Yes
	Increase	38%	(l)	
PRQ	Decrease	68%	(m)	Yes
	Increase	34%	(l)	
QRS	Decrease	56%	(l)	No
	Increase	44%		
P-H	Decrease	50%	(l)	No
	Increase	50%		
R-H	Decrease	56%	(l)	No
	Increase	44%		
HR	Decrease	75%	(m)	Yes
	Increase	25%	(l)	
HRV	Decrease	81%	(h)	Yes
	Increase	29%	(l)	

(*m*): Low

(*l*): Low

(*h*): High

The summary of the percentage of sensitivity for both analysis is described in Table 6 and Table 7. The sensitivity for the statistical analysis presented in Table 6 shows that HR segment shows sensitivity in increasing pattern while QRS and R-H have no sensitivity value because there are no significant changes recorded for the status in the analysis. HR and HRV segment has the highest sensitivity value of 83.3 and 66.7% respectively for status (*h*). More so, ST, QT and PRQ have 50, 66.7 and 50% sensitivity value respectively for decreasing pattern. However, QT and PRQ have sensitivity score for increasing pattern. Furthermore, the sensitivity for spectral analysis is given in Table 7, it is computed by considering all the ECG segments from Table 5. The sensitivity value for decreasing pattern status (*m*) is 44.4%, which is higher than the sensitivity of status (*h*) which is 11.2%. The sensitivity score is not computed for increasing pattern because there is no change in the status for an increasing pattern.

**Table 6:** Sensitivity for statistical analysis

ECG	Pattern	Statistical analysis status		
		(*l*)	(*m*)	(*h*)
ST	Decrease	33.3%	50%	16.7%
QTC	Decrease	66.7%	33.3%	0.0
QT	Decrease	33.3%	66.7%	0.0
	Increase	83.3%	0.0	16.7%
PRQ	Decrease	50%	50%	0.0
	Increase	83.3%	0.0	16.7%
QRS	Equality			
P-H	Decrease	33.3%	66.7%	0.0
R-H	Equality			
HR	Increase	16.7%	0.0	83.3%
HRV	Decrease	0.0%	33.3%	66.7%

(*m*): Low

(*l*): Low

(*h*): High

**Table 7:** Sensitivity for spectral analysis

ECG		Statistical analysis status		
	Pattern	(*l*)	(*m*)	(*h*)
ALL SEGMENTS	Decrease	44.4%	44.4%	11.2%

(*m*): Low

(*l*): Low

(*h*): High

## Conclusions

In this paper, we explore the changes in ECG segments associated with blood glucose from 16 participants. We conducted two experiments for each participant for 2 days. Oral glucose tolerance test is performed on the first day, where the participant is required to consume 75 g of anhydrous glucose solution during the experiment. On the second day, no glucose is consumed to serve as a control experiment. During each experiment, ECG data is collected continuously, while blood is taken at an interval from the tip of the finger. Statistical and spectral analysis is performed on the acquired ECG data to investigate segments that change with glucose for the possibility of achieving non-invasive blood glucose measurement. For the statistical analysis, we computed the boxplot for the ECG data to compare the difference between the ECG data when glucose is consumed and when glucose is not consumed. HR and HRV segments recorded 81% for change in the first quartile, median and mean parameters. While HR has 88% change in the third quartile and 81% in the mode parameters, HRV segment has 81% change in the third quartile and 75% change for the mode parameter. This result shows that HR and HRV segments are significantly influenced by glucose. Another significant result was found in the ST segment, which includes 63, 81 and 75% change for median, third quartile and mode parameters respectively. More so, the QT segment recorded 81, 69 and 75% change for median, mean and third quartile respectively. PRQ and P-H segment obtained some results that make it useful to be combined with other parameters for the possibility of achieving non-invasive blood glucose monitoring.

For spectral analysis, the result shows that HR and HRV segment are significantly affected by glucose because they have the highest value of change pattern of 75 and 81% respectively. While ST, QT and PRQ segments have 75, 62 and 68% change respectively. Therefore, from both analysis, the following segments: HR, HRV, ST, QT, PRQ and P-H should be considered for the possibility of achieving non-invasive blood glucose monitoring with ECG. Moreover, possible future works we intend to consider based on the results obtained in this paper include; investigating the pattern in HRV and HR segment from the participants that deviated from the expected general pattern. Also, to further corroborate the result obtained in this paper, we intend to carry out similar analysis with advance technique on data from diabetes patients. Also, there are heart conditions, such as cardiac arrhythmia that can affect the state or shape of ECG signals. There is a need to investigate how these conditions can be handled or put into consideration to achieve the possibility of non-invasive blood glucose monitoring.

## Supplementary information

**Additional file 1.** The result of the correlation relationship between ECG segments. The correlation is a cross matrix, where the vertical column is ST, QTC and QT segments and the horizontal row are ORQ, P-H, QRS, R-H, HRV and HR segments.

## Data Availability

The datasets used and/or analyzed during the current analysis are available from the corresponding author on reasonable request.

## Abbreviations

BG: Blood glucose
CSV: Comma-separated value
ECG: Electrocardiogram
G: Glucose
HR: Heart rate
HRV: Heart rate variability
IDF: International Diabetes Federation
IRB: Internal Review Board
NG: Non-glucose
OGTT: Oral glucose tolerance test
PSD: Power spectra density
SEF: Suction effusion fluid
WHO: World Health Organization
