# Supplementary material for: Statistical and spectral analysis of ECG signal towards achieving non-invasive blood glucose monitoring
Source: BMC Med Inform Decis Mak. 2019 Dec 19;19(Suppl 6):266. doi: 10.1186/s12911-019-0959-9 (PMC6921435; doi:10.1186/s12911-019-0959-9)
Supplement: Supplementary file 1 — Additional file 1. The result of the correlation relationship between ECG segments. The correlation is a cross matrix, where the vertical column is ST, QTC and QT segments and the horizontal row are ORQ, P-H, QRS, R-H, HRV and HR segments. [file 12911_2019_959_MOESM1_ESM.docx]

Correlation results between ECG segments:

|  | **ST** | **QTC** | **QT** |
| --- | --- | --- | --- |
| **PRQ** |  |  |  |
| **P-H** |  |  |  |
| **QRS** |  |  |  |
| **R-H** |  |  |  |
| **HRV** |  |  |  |
| **HR** |  |  |  |

P: Positive correlation

N: Negative correlation

Z: No (Zero) correlation
